# Altered Expression of GABA-Related Genes in Schizophrenia: Insights from Meta-Analyses of Brain and Blood Samples and iPSC-Derived Organoids

**DOI:** 10.31083/AP43531

**Published:** 2026-02-25

**Authors:** Yuval Singer, Assif Yitzhaky, Libi Hertzberg

**Affiliations:** ^1^School of Medicine, Gray Faculty of Medical & Health Sciences, Tel Aviv University, 69978 Tel Aviv, Israel; ^2^Department of Physics of Complex Systems, Weizmann Institute of Science, 7610001 Rehovot, Israel; ^3^Shalvata Mental Health Center, 45100 Hod Hasharon, Israel

**Keywords:** schizophrenia, gene expression, glutamic acid decarboxylase, somatostatin, parvalbumin

## Abstract

**Background:**

Schizophrenia, one of the most disabling mental disorders, affects approximately seven per 1000 individuals worldwide and has an estimated heritability of around 80%; however, its pathophysiology remains incompletely understood. The disorder has been linked to dysregulation of multiple neurotransmitter systems, including dopamine, serotonin, γ-aminobutyric acid (GABA), and glutamate. GABA, the primary inhibitory neurotransmitter in the central nervous system, is synthesized by the enzymes glutamic acid decarboxylase 67 (GAD67) and glutamic acid decarboxylase 65 (GAD65), encoded by the *GAD1* and *GAD2* genes, respectively. The genes (*SST*) and parvalbumin (*PVALB*) encode somatostatin and parvalbumin, which are characteristic markers of specialized GABAergic interneuron subpopulations involved in maintaining excitatory–inhibitory balance and supporting cortical circuit function. While reduced *GAD1* expression has been consistently reported in schizophrenia, findings regarding *GAD2* expression have been inconsistent.

**Methods:**

In this study, we examined the expression of *GAD1*, *GAD2*, *SST*, and *PVALB* across three biological levels: postmortem brain tissue, peripheral blood samples, and patient-derived induced pluripotent stem cell (iPSC)-derived brain organoids, compared with healthy controls. The meta-analysis of brain tissue included seven independent datasets (295 samples: 151 individuals with schizophrenia and 144 healthy controls) and was conducted in accordance with the Preferred Reporting Items for Systematic Reviews and Meta-Analyses (PRISMA) guidelines. Patient-derived iPSC organoids were used to investigate early neurodevelopmental alterations, while a separate meta-analysis of peripheral blood gene expression included 293 samples (160 schizophrenia, 133 controls) to explore biomarker potential.

**Results:**

Both *GAD1* and *GAD2* were significantly downregulated in postmortem brain samples (meta-analytic effect sizes <–0.5) and in iPSC-derived organoids, supporting the hypothesis that reduced expression of these genes emerges prior to clinical onset and may contribute to disease development. In contrast, decreased expression of *SST* and *PVALB* was observed in brain tissue but not in organoids, suggesting that alterations in these interneuron markers may occur at later stages of the disease. Notably, reduced *PVALB* expression was also detected in peripheral blood samples, indicating its potential utility as a peripheral biomarker for schizophrenia.

**Conclusions:**

Further studies are required to clarify the causal role of reduced GABAergic activity in schizophrenia pathogenesis and to evaluate the clinical relevance of *PVALB* expression for diagnosis and treatment monitoring.

## Main Points

1. glutamic acid decarboxylase 1 (GAD1) and GAD2 were downregulated in both postmortem brains and organoids derived from patients with schizophrenia, suggesting a neurodevelopmental origin.

2. Somatostatin (SST) and parvalbumin (PVALB) were reduced in brain tissue but trended upward in organoids, indicating their downregulation may arise later in disease progression.

3. PVALB downregulation in blood suggests its potential as a biomarker.

## 1. Introduction and Scientific Background

Schizophrenia is a complex psychiatric disorder, affecting roughly 0.7% of the people worldwide [1]. The underlying cause of the disease remains unclear. Still, it is widely acknowledged to stem from an intricate interplay of genetic and environmental factors, with genetics playing a substantial role, accounting for around an 80% heritability [2, 3]. Genetic studies, such as genome-wide association studies (GWAS), have identified hundreds of common single-nucleotide polymorphisms (SNPs) associated with schizophrenia [4]. The majority of these schizophrenia associated SNPs are non-coding variants situated within regulatory DNA elements [5, 6]. This pattern strongly implies that the interplay between genetic variations and schizophrenia phenotypes is predominantly mediated through gene expression [7]. Therefore, the study of gene expression and the identification of differentially expressed genes are critical to improving our understanding of the biological basis of schizophrenia.

Identifying distinct abnormalities in schizophrenia is challenging due to its involvement in multiple biochemical pathways. The disease is linked to neurotransmitters like dopamine, serotonin, norepinephrine, gamma-aminobutyric acid (GABA), and glutamate [8].

GABA is the primary inhibitory neurotransmitter and plays an essential role in neural network oscillations, balancing excitation and inhibition, maturation of neural circuitry, and neuronal differentiation [9].

GABA has a regulatory effect on dopamine activity, and a decline in GABAergic activity could lead to hyperactivity of dopaminergic neurons, which is thought to be involved in schizophrenia [10, 11, 12].

GABA is synthesized by the enzymes glutamic acid decarboxylase (GAD) 67 and GAD65, encoded by the *GAD1* and *GAD2* genes, respectively. *GAD1* is primarily expressed in the brain and is predominantly located in the cytoplasm, where it supports continuous GABA production for cell metabolism and tonic, non-vesicular GABA release. In contrast, *GAD2* is expressed both in the brain and the pancreas and is preferentially localized in axon terminals, associated with synaptic vesicles, where it plays a critical role in activity-dependent, vesicular GABA release during intense synaptic activity [13, 14].

In genetic studies, there is evidence of a notable association between variants in the *GAD1* gene and schizophrenia, which supports the hypothesis that it plays a causal role in the development of schizophrenia [15, 16]. However, the latest GWAS did not detect a significant association between *GAD1* or *GAD2* and schizophrenia, as demonstrated by the RICOPILI tool [4, 15].

Multiple studies have reported downregulation of *GAD1* in schizophrenia, evidenced through the analysis of postmortem brain samples for messenger RNA (mRNA) and protein levels [16, 17]. Notably, a review of 25 postmortem studies shows a consistent reduction of *GAD1* gene and the encoded GAD67 protein in the dorsolateral prefrontal cortex (DLPFC) [17, 18, 19]. For example, a postmortem study measured both *GAD1* mRNA and GAD67 protein levels in the same DLPFC samples from individuals with schizophrenia, finding coordinated significant reductions in both measures. In contrast, GAD65 protein levels did not show a significant reduction [17]. Similar concordant reductions in *GAD1* mRNA and GAD67 protein were reported in a second study of schizophrenia DLPFC samples [18].

GABAergic neurons can be subdivided into distinct types based on the specific molecular markers they express. Among these are parvalbumin (PVALB) expressing interneurons, characterized by the calcium-binding protein parvalbumin, and somatostatin (SST) expressing interneurons, marked by the neuropeptide somatostatin. These interneuron types differ significantly in their anatomical features, connectivity, and electrophysiological properties. SST interneurons predominantly target dendritic regions and enhance excitatory input processing, while PVALB interneurons specialize in providing rapid, synchronized inhibition at the soma and proximal dendrites, thus facilitating faster neural dynamics within cortical circuits [20, 21].

*GAD1* expression was reduced in PVALB-expressing prefrontal cortex (PFC) interneurons, where around 50% lacked detectable levels of *GAD1* in individuals with schizophrenia [22]. Interestingly, both SST and PVALB-expressing GABA neurons showed downregulated expression of SST and PVALB, respectively, in the DLPFC of individuals with schizophrenia [23]. In addition to the DLPFC, additional brain regions were shown to have downregulated expression of *GAD1* [12, 19]. Fewer studies explored the levels of *GAD2* and the encoded protein GAD65, and the findings were less consistent, as summarized in Table 1 (Ref. [17, 18, 24]) and Table 2 (Ref. [11, 12, 16, 17, 18, 22, 25, 26, 27, 28, 29]).

**Table 1.** **Studies examining GAD65 and GAD67 protein expression in postmortem brain tissue from individuals with schizophrenia**.

Protein	Examination Area	Higher/Lower Expression	SZ	CNT	Reference
GAD67 (encoded by GAD1)	PFC and cerebellum	↓	15	15	[17]
	DLPFC	↓	19	19	[18]
	DLPFC	↓	20	20	[24]
GAD65 (encoded by GAD2)	PFC and cerebellum	↔	15	15	[17]

Abbreviations: DLPFC, dorsolateral prefrontal cortex; PFC, prefrontal cortex; SZ, schizophrenia; CNT, control. ↓, lower expression; ↔, no differential expression.

**Table 2.** **Studies of GAD1 and GAD2 transcript levels in postmortem brain tissue from schizophrenia and control groups**.

Gene	Examination Area	Higher/Lower Expression	SZ	CNT	Reference
*GAD1*	DLPFC	↓	42	42	[18]
	DLPFC	↓	14	14	[25]
	DLPFC, ACC, primary motor and primary visual cortices	↓	12	12	[26]
	cerebellum	↓	15	15	[11]
	PFC	↓	15	15	[22]
	DLPFC	↓	37	37	[16]
	PFC and cerebellar expression	↓	15	15	[17]
	OFC, STG, caudate, putamen, nucleus accumbens, medial dorsal thalamus and anterior thalamus	↓	14	15	[12]
	PFC	↓	10	10	[27]
	DLPFC	↓	41	43	[28]
*GAD2*	DLPFC	↓	41	43	[28]
	PFC and cerebellar expression	↔	15	15	[17]
	DLPFC, ACC, primary motor and primary visual cortices	↓	12	12	[26]
	cerebellum	↓	15	15	[11]
	ACC and PFC	↔	12	12	[29]

Abbreviations: DLPFC, dorsolateral prefrontal cortex; ACC, anterior cingulate cortex; PFC, prefrontal cortex; OFC, orbitofrontal cortex; STG, superior temporal gyrus. ↓, lower expression; ↔, no differential expression.

It should be noted that postmortem studies cannot determine whether differential expression is a causative factor or a consequence of the development of schizophrenia. In this context, recent technological advancements have significantly benefited *in vitro* organoid preparations, advancing developmental neuroscience [30]. Brain organoids, which are self-assembled three-dimensional aggregates derived from pluripotent stem cells, mimic embryonic human brain structures. This makes them valuable models for studying brain development and disorders [31]. Research on schizophrenia using patient-derived cerebral organoids has uncovered unique gene expression profiles, for example, in genes related to synaptic function and the regulation of excitation-inhibition balance, including significant downregulation of *GAD1* and *GAD2* [32].

In conclusion, while previous findings regarding *GAD1* expression were consistent, there are inconsistencies regarding *GAD2* gene expression in tissue samples from subjects with schizophrenia. Moreover, the interplay between these genes remains unclear.

We conducted a systematic participant data meta-analysis following the PRISMA 2020 guidelines (**Supplementary Material-PRISMA_checklist**) [33]. Publicly available gene expression datasets from brain samples were included to systematically calculate the differential expression of *GAD1*, *GAD2*, as well as *SST* and *PVALB*, and their correlation patterns in different brain regions of patients with schizophrenia. Additionally, we examined their expression in organoids derived from patients, offering insights into early neurodevelopmental stages, and in blood samples from patients to assess their potential as biomarkers.

## 2. Methods

### 2.1 Selection of Gene Expression Datasets for Participant Data Meta-Analysis

We identified eligible transcriptomic datasets by querying two major repositories containing human postmortem brain samples gene expression data: the Gene Expression Omnibus (GEO) of the National Center for Biotechnology Information (NCBI) and the Stanley Medical Research Institute (SMRI) Array collection. The search combined terms related to schizophrenia, brain tissue, and transcriptomic profiling (“schizophrenia”, “human”, “brain samples”, “gene expression”), aiming to capture case-control datasets in which individuals with schizophrenia were compared to unaffected controls and for which processed genome-wide expression matrices were available.

**Fig. 1.**
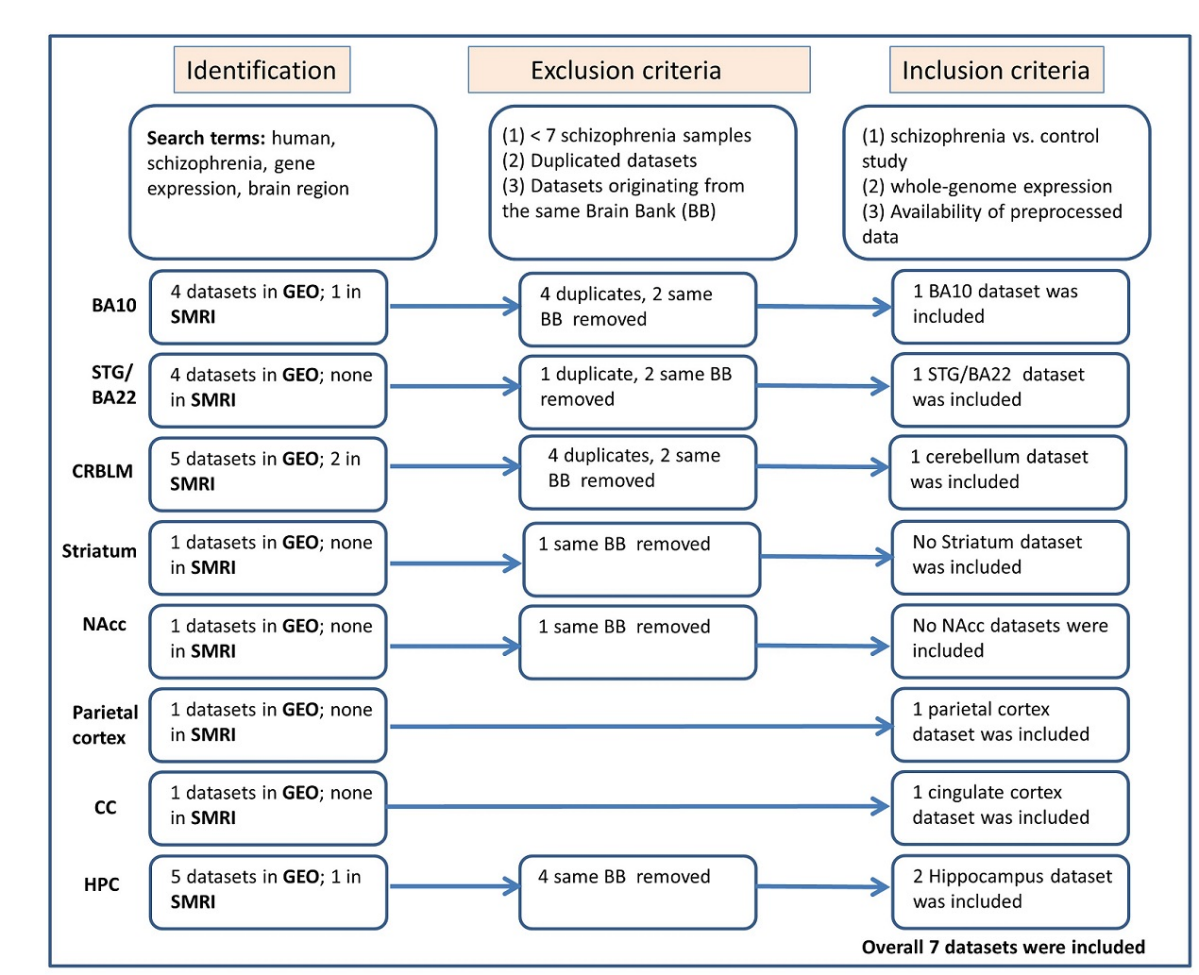
**Workflow of participant data meta-analysis following PRISMA guidelines [33]**. Abbreviations: SMRI, Stanley Medical Research Institute; GEO, Gene Expression Omnibus; CMC, CommonMind Consortium; BA, Brodmann area; STG, superior temporal gyrus; NAcc, Nucleus Accumbens; CC, Cingulate Cortex; HPC, Hippocampus; PRISMA Preferred Reporting Items for Systematic reviews and Meta-Analyses.

The selection process, illustrated in Fig. 1 (Ref. [33]) and informed by PRISMA guidelines [33], focused on studies sampling the following brain regions: Brodmann area 10 (frontal cortex), Brodmann area 22/superior temporal gyrus, cerebellum, parietal cortex, anterior cingulate cortex, hippocampus, striatum, and nucleus accumbens. To ensure sufficient statistical power and comparability across studies, datasets were required to include clear diagnostic classification, essential metadata describing tissue quality (such as age, sex, brain pH, and postmortem interval), and compatible expression profiling platforms. See **Supplementary Material** for a detailed description of the search strategy.

We also analyzed three blood-derived gene expression datasets and one iPSC-derived cerebral organoid dataset (Table 3, Ref. [32, 34, 35, 36, 37, 38, 39, 40, 41, 42, 43]). These datasets were selected based on availability from previous analyses rather than through a systematic selection process.

**Table 3A.** **Overview of the brain datasets incorporated into the participant-data meta-analysis**.

Brain samples datasets								
Accession	Publication	Brain region; Brain bank	# SZ	# CNT	Platform	Mean Age (standard dev.)	Mean PMI (standard dev.)	Mean pH (standard dev.)
GDS4523	[34]	BA10; CCHPC	27	23	HG U133 Plus 2.0	SZ: 73 (15)	SZ: 8.2 (7)	SZ: 6.1 (0.2)
			19M:8F	12M:11F		CNT: 69 (22)	CNT: 10 (4)	CNT: 6.5 (0.3)
						*p* = 0.45	*p* = 0.3	*p* = 8 × 10^-6^
GSE37981	[35]	STG Pyramid.; HBTRC	9	8	U133 X3P Array	SZ: 67 (20)	SZ: 17 (5)	Not provided
			4M:5F	4M:4F		CNT: 67 (21)	CNT: 18 (3)	
						*p* = 0.99	*p* = 0.71	
GDS1917	[36]	CRBLM; Maryland	13	14	U133 Plus 2.0 Array	SZ: 46 (12)	SZ: 12.8 (5)	Not provided
			13M:0F	14M:0F		CNT: 43 (10)	CNT: 15.6 (6)	
						*p* = 0.5	*p *= 0.18	
GSE35978	[37]	Parietal cortex; SMRI	51	45	Gene 1.0 ST Array	SZ: 43 (10)	SZ: 31 (16)	SZ: 6.4 (0.3)
			37M:14F	31M:14F		CNT: 46 (9)	CNT: 27 (12)	CNT: 6.5 (0.3)
						*p* = 0.14	*p *= 0.17	*p *= 0.015
GSE80655	[38]	ACC; Pritzker	23	24	Illumina HiSeq 2000	SZ: 43 (9)	SZ: 21 (9)	SZ: 6.8 (0.2)
			20M:3F	21M:3F		CNT: 50 (13)	CNT: 22 (7)	CNT: 6.9 (0.1)
						*p *= 0.043	*p* = 0.62	*p *= 0.044
GSE53987	[39]	HPC; Pittsburgh	15	18	HG U133 Plus 2.0	SZ: 46 (9)	SZ: 19 (7)	SZ: 6.4 (0.3)
			9M:6F	9M:9F		CNT: 48 (11)	CNT: 19 (5)	CNT: 6.6 (0.2)
						*p *= 0.49	*p *= 0.99	*p* = 0.055
GSE138082	[40]	HPC CA3; Dallas	13	12	Illumina HiSeq 2500	SZ: 54 (11)	SZ: 23 (7)	Not provided
			9M:4F	9M:3F		CNT: 56 (9)	CNT: 20 (4)	
						*p *= 0.59	*p *= 0.2	
			Overall: 151	Overall: 144				

Abbreviations: PMI, postmortem interval; BA, Brodmann area; CCHPC, Charing Cross Hospital Prospective Collection; STG, superior temporal gyrus; CRBLM, cerebellum; SMRI, Stanley Medical Research Institute; ACC, anterior cingulate cortex; HPC, hippocampus; M, male; F, female; pyramid, pyramidal neurons; Pritzker, Pritzker Neuropsychiatric Disorders Research Consortium; Pittsburgh, Brain Tissue Donation Program at the University of Pittsburgh; Dallas, Dallas Brain Collection; Maryland, Maryland Brain Collection; HBTRC, Harvard Brain Tissue Resource Center; CA, Cornu Ammonis.

**Table 3B.** **Overview of the blood and organoids datasets incorporated into the participant-data meta-analysis**.

Blood samples datasets					
Accession	Publication	Cell type	# SZ	# CNT	Platform
GSE38481	[41]	PBMC	41	29	Affymetrix Human Genome U133 Plus 2.0 Array
GSE18312	[42]	Whole blood	13	8	Affymetrix Human Exon 1.0 ST Array
GSE38484	[43]	Whole blood	106	96	Illumina HumanHT-12 V3.0 expression beadchip
			Overall: 160	Overall: 133	
Organoids derived from iPSCs dataset					
Accession	Publication	Samples type	# SZ	# CNT	Platform
GSE133534	[32]	iPSC derived cerebral organoids	8	8	Illumina NovaSeq 6000

Abbreviations: PBMC, peripheral blood mononuclear cells; ST, sense target; iPSC, induced pluripotent stem cell. Two-sided *t*-tests were used to compare mean age, PMI (expressed in hours), and pH between SZ and CNT groups for each dataset.

Extracted characteristics for each dataset included the sampled region, number of cases and controls, profiling platform, and the available metadata noted in Table 3. Additional technical details, such as the normalization and outlier-filtering procedures applied, are provided in the **Supplementary Material**. Importantly, dataset inclusion was finalized prior to performing differential expression analyses of the target GABAergic-related genes- *GAD1*, *GAD2*, *PVALB*, and *SST*- to eliminate the possibility of selection bias toward datasets showing effect for these genes.

### 2.2 Gene Expression Meta-Analysis

To evaluate expression differences, we performed a participant-level meta-analysis for each gene. For every dataset, we computed Hedges’ g [44, 45] (the standardized mean difference between schizophrenia and control groups) along with its confidence interval. Positive values indicated higher expression in schizophrenia. These calculations were done with the “metacont” function of the meta package in R (version 3.6.1, R Foundation for Statistical Computing, Vienna, Austria) [46, 47].

Because of heterogeneity in platforms and study designs, effect sizes were pooled using a random-effects model [45]. This approach incorporates both the magnitude and direction of expression changes, producing more reliable estimates of underlying biological effects [47].

### 2.3 Analysis of Potential Confounding Factors

We also examined whether variables such as tissue pH, postmortem interval (PMI), and participant age might influence expression differences. For each gene, we fitted a multiple linear regression model [48] in MATLAB (version R2020b, ’meta’ package version: 4.9-5. “fitlm” function, default parameters, The MathWorks, Inc., Natick, MA, USA), with diagnosis as the main predictor and the above factors as covariates. When available, RNA integrity number (RIN) and cumulative antipsychotic exposure (quantified as lifetime fluphenazine-equivalent dose, mg) were also included. For each gene, the diagnosis coefficient was statistically tested for being nonzero, implying an effect for schizophrenia, beyond any other effect of the covariates. This produced a t-statistic and a corresponding *p*-value.

### 2.4 Heterogeneity Measures

To assess heterogeneity across the datasets included in the meta-analysis, we quantified between-study variability using three complementary measures: Cochran’s Q, I^2^, and τ^2^. Cochran’s Q provides a formal statistical test for heterogeneity by evaluating whether the observed variability in effect sizes exceeds what would be expected by chance alone, based on the weighted sum of squared deviations from the pooled effect size [49]. To quantify the degree of inconsistency that could be attributed to genuine between-study variation rather than chance, the I^2^ (Inconsistency) statistic was calculated, expressing the proportion of total variation due to heterogeneity [50]. Furthermore, the magnitude of the between-study variance was estimated using τ^2^, which provided the essential variance component for the subsequent random-effects model employed in the meta-analysis [51]. Together, these metrics allowed us to evaluate the extent and nature of heterogeneity among the included datasets and to interpret the robustness of the meta-analytic findings.

## 3. Results

### 3.1 GAD1, GAD2, SST, and PVALB are Downregulated in Brain Samples of Patients With Schizophrenia

**Fig. 2.**
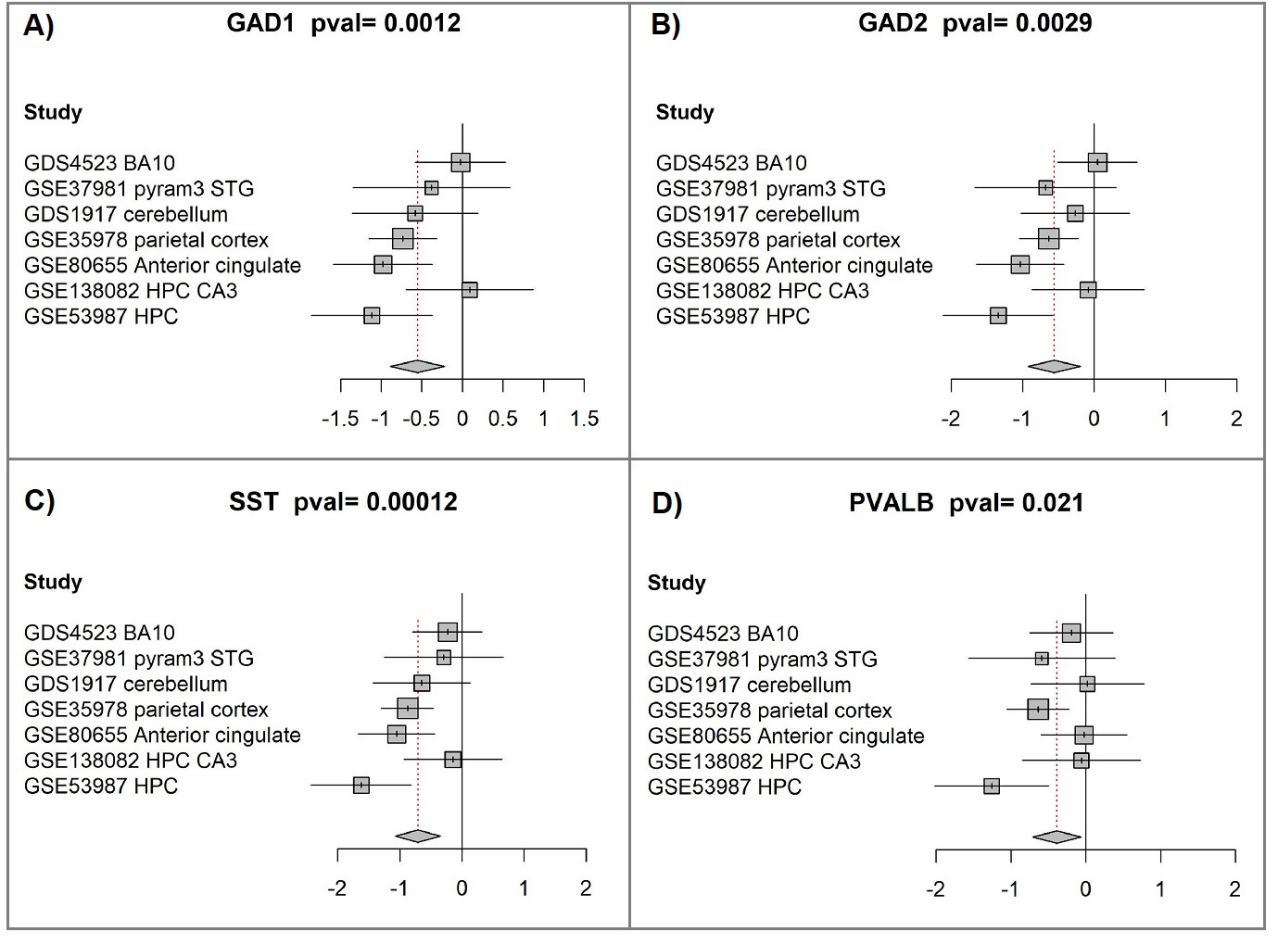
**Participant-level meta-analysis indicates reduced expression of GAD1, GAD2, SST, and PVALB in schizophrenia brain tissue**. (A) *GAD1* meta-analysis. Forest plots were produced with the “forest” function in the R package meta (version 4.9-2). For each study included, the plot compares expression values between the schizophrenia and control groups. Squares mark standardized mean differences [46, 47]; their size corresponds to the study’s weight, determined primarily by sample number. Horizontal bars represent the 95% confidence intervals, while the vertical line indicates no significant group difference. Positive values indicate higher expression in schizophrenia, whereas negative values represent reduced expression. The diamond at the bottom summarizes the overall effect, with its center indicating the pooled estimate and its width the 95% confidence range. The *p*-value for the combined analysis appears in the figure title. (B) *GAD2* meta-analysis. (C) *SST* meta-analysis. (D) *PVALB* meta-analysis.

We have performed a participant-data systematic meta-analysis of *GAD1*, *GAD2*, *SST*, and *PVALB* genes’ expression in 295 brain samples (151 individuals with schizophrenia vs. 144 healthy controls), integrating seven independent gene expression datasets (Table 3). The four genes were found to be significantly downregulated in brain samples of individuals with schizophrenia (Fig. 2, Ref. [46, 47]; Table 4, Ref. [45]).

**Table 4.** **Summary of the meta-analytic differential expression results in brain tissue**.

#	Gene symbol	Random effects Hedges	Lower	Upper	*p*-value	τ2	I2	Q	Q *p*-value
1	GAD1	–0.55	–0.89	–0.22	0.0012	0.3	0.45	11	0.09
2	GAD2	–0.56	–0.93	–0.19	0.0029	0.36	0.54	13	0.042
3	PVALB	–0.38	–0.71	–0.06	0.021	0.28	0.43	10	0.11
4	SST	–0.71	–1.07	–0.35	0.00012	0.34	0.51	12	0.055

Standardized effect sizes were calculated under a random-effects model using Hedges’ g [45]. Negative values (blue) reflect decreased gene expression in schizophrenia relative to controls, whereas positive values (red) indicate higher expression in schizophrenia than in controls. In the present table, only negative effect sizes were observed; therefore, no red shading is shown. The shading intensity corresponds to the magnitude of the effect. Columns 4 and 5 present the bounds of the 95% confidence interval, and the associated *p*-values are provided in column 6. Measures of between-study variability (I^2^ and τ^2^) and the Cochran’s Q statistic with its *p*-value are listed in columns 7 through 10.

### 3.2 GAD1, GAD2, SST, and PVALB Expression Levels are Significantly Correlated in Brain Samples of Patients With Schizophrenia

**Fig. 3.**
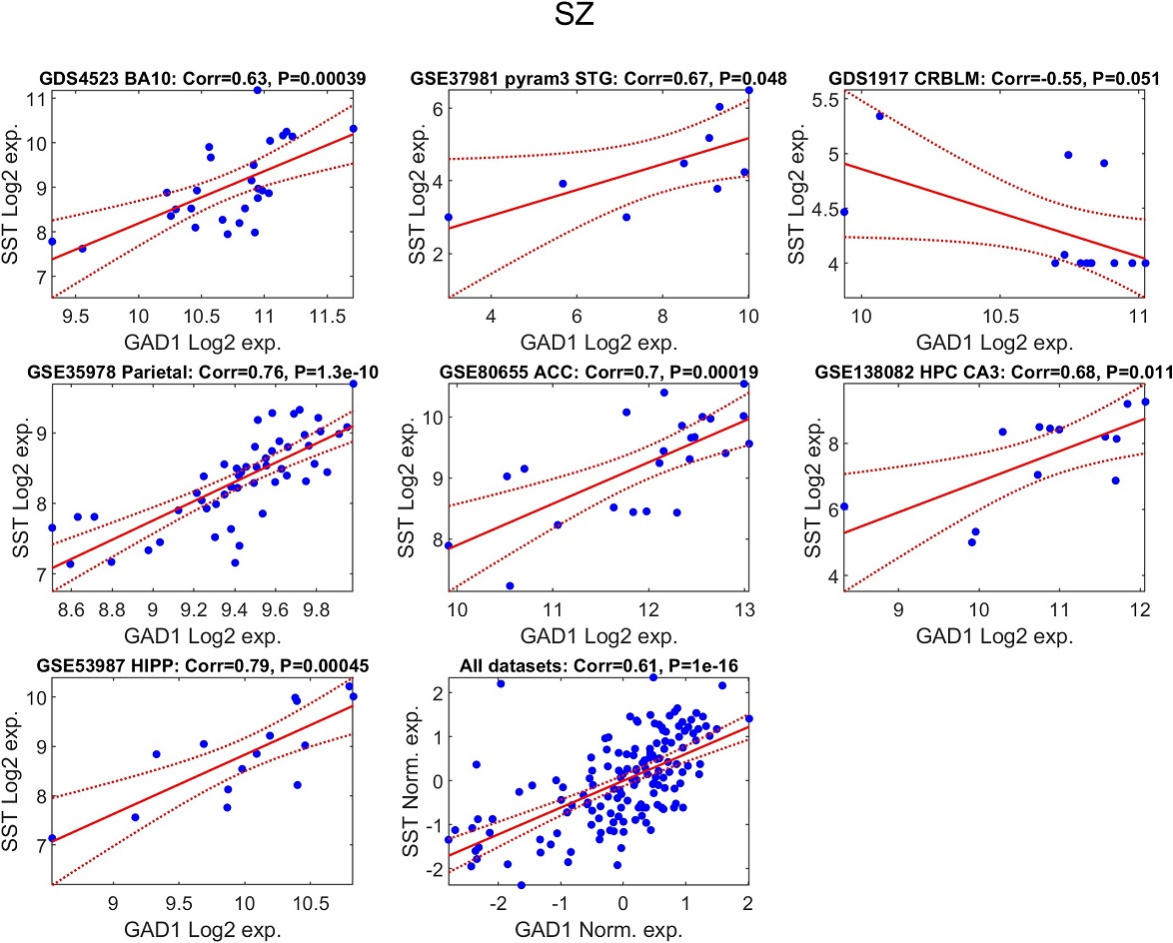
**Positive association between *GAD1* and *SST* transcript levels in schizophrenia**. For each of the seven independent datasets, the relationship between *SST* and *GAD1* expression in schizophrenia samples is illustrated by a scatter plot. Each point corresponds to one individual with schizophrenia. A fitted regression line is displayed in red, with its 95% confidence region shown as dashed red boundaries. The Pearson correlation coefficient and its significance value are indicated in the title of each panel. In addition, a combined dataset analysis is shown after standardizing gene expression within each study (mean = 0; standard deviation = 1), enabling a joint evaluation of this expression relationship across cohorts.

To examine the existence of common regulation in patients with schizophrenia, we performed Pearson correlation analysis between the expression levels of each pair of genes, in each dataset separately among the schizophrenia samples. All four genes were found to have significantly correlated expression levels (**Supplementary Fig. 1**). For example, *GAD1* and *SST* (Fig. 3) show a significant positive correlation (combined data corr. = 0.61, *p*-value = 1 × 10^-16^). The strong positive correlations are consistent with our meta-analytic findings and suggest that the results are unlikely to be driven by random variation.

### 3.3 Examination of Potential Confounding Factors

To evaluate whether factors such as age, brain pH, postmortem interval (PMI), RNA integrity number (RIN), and antipsychotic exposure (quantified as lifetime fluphenazine-equivalent dose, mg) influenced the results, we applied linear regression models that included these variables as covariates across the seven available datasets (see Methods). The corresponding outcomes are provided in **Supplementary Table 1**. Not all studies contained complete information for every variable. To summarize, we calculated weighted mean t-statistics for *GAD1*,* GAD2*,* SST*, and *PVALB*. After adjusting for the covariates, all four genes showed consistent downregulated expression (**Supplementary Table 1**; mean t-statistics: –1.01, –0.98, –1.54, –0.74, respectively).

### 3.4 Downregulation of PVALB in Blood-Derived Samples

**Fig. 4.**
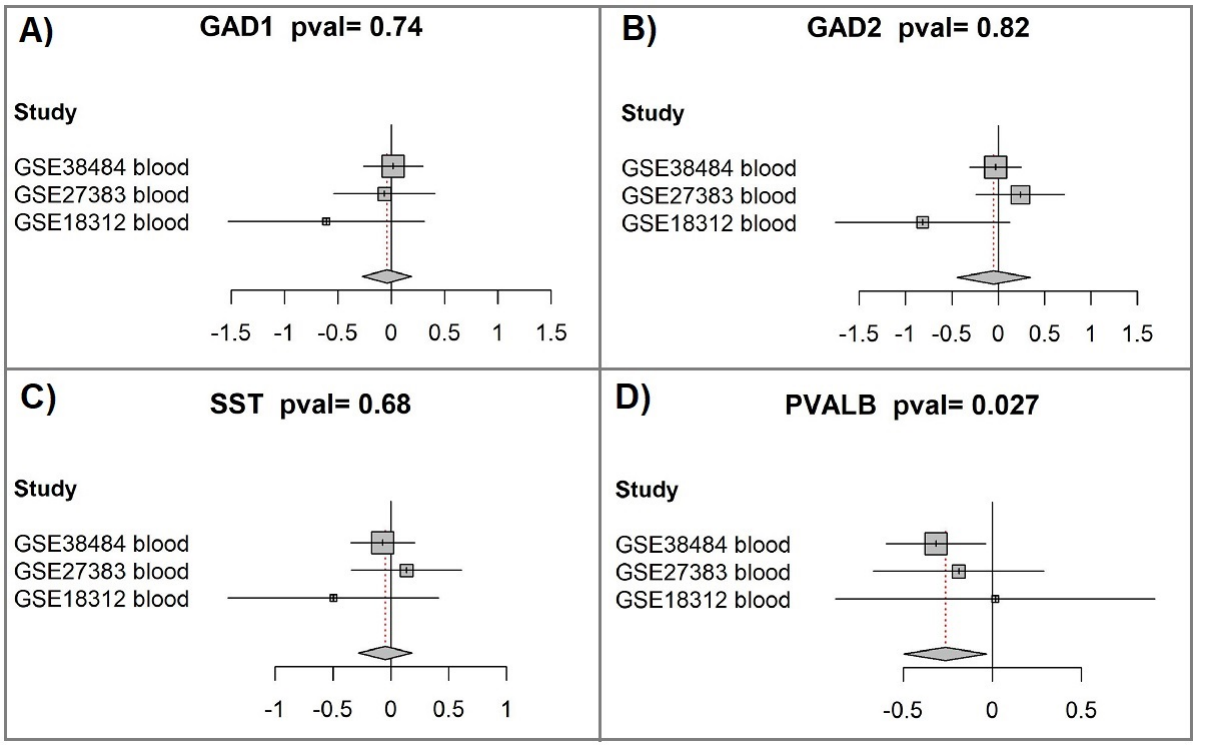
**Meta-analysis of peripheral blood samples indicates reduced *PVALB* expression in schizophrenia**. (A) *GAD1* meta-analysis. Forest plots were produced with the “forest” function from the R package meta (version 4.9-2) [46, 47]. The plots compare gene expression between schizophrenia patients and controls across the included studies. Each square marks the standardized mean difference [45] for an individual dataset, with its size reflecting the relative weight contributed, largely determined by sample size. Horizontal bars denote 95% confidence intervals, while the vertical line corresponds to no difference between groups. Positive values indicate higher expression in schizophrenia, and negative values indicate reduced expression. The diamond at the bottom represents the pooled result, with its midpoint giving the overall effect and its width the 95% confidence range. The *p*-value for the combined test is shown in the figure title. (B) *GAD2* meta-analysis. (C) *SST* meta-analysis. (D) *PVALB* meta-analysis.

We next investigated whether *GAD1*, *GAD2*, *SST*, and *PVALB* exhibited altered expression in blood from patients with schizophrenia, aiming to explore their potential as biomarkers. A participant-data meta-analysis was carried out on three publicly available datasets, comprising 293 peripheral blood samples (160 schizophrenia and 133 controls), see Table 3. Additional dataset details, including normalization and preprocessing methods, are available in the **Supplementary Material**. The analysis showed significant downregulation of *PVALB* in schizophrenia blood samples (Hedges’ g effect size = –0.26, CI: –0.5 to –0.03, *p* = 0.027; Fig. 4D), highlighting its possible utility as a biomarker, whereas *GAD1*, *GAD2*, and *SST* did not differ significantly between groups (Fig. 4A–C (Ref. [45, 46, 47]); **Supplementary Table 2**).

### 3.5 Reduced GAD1 and GAD2 Expression in Cerebral Organoids Derived From Schizophrenia iPSCs

**Fig. 5.**
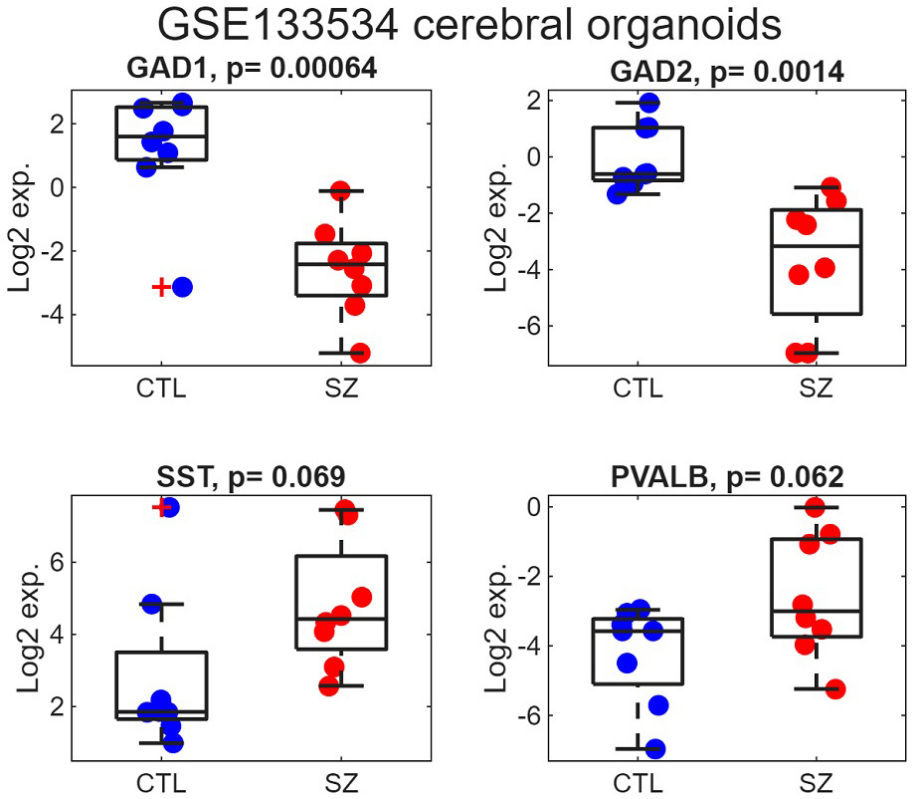
**Reduced expression of GAD1 and GAD2 in iPSC-derived cerebral organoids from schizophrenia donors**. Boxplots display the distribution of Log2 transcript levels for each gene in the GSE133532 organoid dataset, comparing control samples (blue) and schizophrenia samples (red). Individual points represent the Log2 expression measured for each subject. The central line of each box shows the median, and the lower and upper box boundaries correspond to the 25th and 75th percentiles. Associated two-sided *t*-test *p*-values are reported in the subplot titles. Observations lying more than 1.5 times the interquartile range beyond the box boundaries are considered outliers and are shown as red crosses. CTL, control.

To explore gene expression in a developmental model, we reanalyzed a publicly available cerebral organoid transcriptome dataset generated from induced pluripotent stem cells of schizophrenia patients and matched controls. Normalized data from GSE133534 [32], which included 16 samples in total (8 cases and 8 controls), were obtained from the GEO repository. Consistent with prior reports [32], both *GAD1* and *GAD2* displayed significant downregulation (Fig. 5; *p* = 0.00064 and 0.0014, respectively). By contrast, *SST* and *PVALB* exhibited a modest tendency toward increased expression in schizophrenia (*p* = 0.069 and 0.062, respectively).

## 4. Discussion

In our study, we conducted a systematic participant data meta-analysis of seven brain samples gene expression datasets (overall 295 samples, 151 schizophrenia vs. 144 controls). Our analysis revealed a marked reduction in *GAD1* (Hedges’ g = –0.55, 95% CI: –0.89 to –0.22, *p* = 0.0012), *GAD2* (Hedges’ g = –0.56, 95% CI: –0.93 to –0.19, *p* = 0.0029), *PVALB* (Hedges’ g = –0.38, 95% CI: –0.71 to –0.06, *p* = 0.021) and *SST* (Hedges’ g = –0.71, 95% CI: –1.07 to –0.35, *p* = 0.00012) in brain samples of individuals with schizophrenia. In addition, *GAD1* and *GAD2* were significantly downregulated in brain organoids derived from individuals with schizophrenia (*p* = 0.00064, 0.0014, respectively), as was previously published [32], while *SST* and *PVALB* showed upregulated expression that did not reach statistical significance (*p* = 0.069, 0.062, respectively). While *GAD1* was previously shown to be downregulated in brain samples of patients with schizophrenia, the results regarding *GAD2* were inconsistent (summarized in Table 2). Our meta-analysis detects significant downregulation of *GAD2* (g = –0.56, *p* = 0.0029) in both cortical and subcortical areas. In addition, as previously observed [32], *GAD1* and *GAD2* showed downregulation in cerebral organoids derived from patients with schizophrenia. This finding supports the hypothesis that gene expression alterations occur during neurodevelopment, rather than solely as a consequence of disease progression.

Beyond the downregulation in brain samples, we also detected downregulation of *PVALB* in blood samples of individuals with schizophrenia (*p* = 0.027), in a meta-analysis of 293 samples (160 schizophrenia and 133 controls). This suggests the *PVALB* gene’s potential role as a biomarker of schizophrenia.

The current study has certain limitations that should be acknowledged. Like other postmortem studies, it captures the neurobiological characteristics at the end of life, thus offering insights solely into the terminal stages. However, by examining also organoids that replicate embryonic human brain structures, we provide insights into gene expression changes occurring during early developmental stages. We note that the downregulation of *GAD1* and *GAD2* has already been detected in the same organoids dataset that we looked at [32], while the expression of *SST* and *PVALB* was not examined. In addition, the dataset we examined has a relatively small sample size (n = 16, comprising 8 cases and 8 controls). This small number of samples may have contributed to the lack of statistical significance when we examined *SST* and *PVALB* differential expression in organoid samples. Another limitation of this work is the relatively small number of included gene expression datasets, which reduces the robustness of our conclusions and constrains our ability to perform more refined analyses. Substantial heterogeneity (I^2^ >50%) in the differential expression of *GAD2* and *SST* was observed, which indicates variation in how these genes were differentially expressed between schizophrenia and controls across datasets (Table 4). For *GAD2*, three of the seven datasets demonstrated significant downregulation in schizophrenia (*p* < 0.05), while the remaining four did not show significant differential expression (Fig. 2B). A similar pattern was observed for SST (Fig. 2C). Notably, two of the four datasets without significant differential expression, GDS1917- cerebellum and GSE37981- pyram3 STG, nonetheless showed downregulation that did not reach statistical significance. This heterogeneity may reflect both technical and biological factors, including differences in platforms, postmortem interval (PMI), pH, medication exposure, and brain regions analyzed. While we could not isolate the impact of each of these factors, they are all plausible contributors to the observed variability.

Moreover, the peripheral blood datasets included in our analysis were heterogeneous, both in terms of sample type (whole blood versus peripheral blood mononuclear cells (PBMCs)) and in gene expression platforms used (Affymetrix versus Illumina arrays). Such variability may have introduced technical and biological noise, potentially contributing to inconsistent findings across genes.

In addition, prolonged exposure to antipsychotics may affect gene expression. To address this bias, we conducted a linear regression analysis that accounted for the potential effect of antipsychotics and additional confounders. While information on the use of antipsychotics was available only for the parietal cortex dataset (GSE35978), all genes consistently exhibited downregulation in schizophrenia, even after accounting for the effects of antipsychotics (**Supplementary Table 1**). The analysis of gene expression of postmortem brain samples is exposed to various technical and biological noise. However, the significant correlation coefficient values we detected between the expression patterns of all four genes (**Supplementary Table 2**) support the results of our meta-analysis and reduce the likelihood of false positives resulting from arbitrary noise. Another limitation is the fact that we measured gene expression levels, which may not accurately reflect the levels of the encoded proteins. Consequently, further investigation is essential to elucidate the implications of the signal we detected in relation to protein levels and activity. Nevertheless, several postmortem studies have demonstrated that decreased *GAD1* mRNA levels correspond to a proportionate reduction in GAD67 protein, supporting the biological relevance of transcript-level findings [17, 18, 27].

## 5. Conclusions

In conclusion, the significant reduction of *GAD1* and *GAD2* expression in both schizophrenia brain samples and organoids supports the hypothesis that decreased expression begins prior to disease onset, potentially contributing to its development. In contrast, the reduced expression of *SST* and *PVALB*, observed in brain samples but not in organoids, likely reflects changes occurring at later stages. The downregulation of *PVALB* in blood samples further suggests its potential as a peripheral biomarker for schizophrenia.

Further research is necessary to clarify the causal role of reduced GABA activity in schizophrenia and to investigate the utility of *PVALB* expression as a biomarker. Validation of *GAD1* and *GAD2* downregulation in independent iPSC-derived organoid models will be essential to confirm reproducibility. Integrating additional molecular layers, such as proteomics, could enhance our understanding of the underlying biological mechanisms. Likewise, assessing *PVALB* expression in larger and more heterogeneous blood cohorts will help establish its robustness as a biomarker for diagnosis, disease monitoring, or treatment response. Together, such efforts would strengthen the translational significance of our findings and help bridge the gap between transcriptomic alterations and clinical applicability.

## Availability of Data and Materials

Gene expression datasets are available through the Gene Expression Omnibus (GEO) database (GDS4523, GSE37981, GDS1917, GSE35978, GSE80655, GSE53987, GSE138082, GSE38481, GSE18312, GSE38484, GSE133534).

## Supplementary Material

Supplementary material associated with this article can be found, in the online version, at https://doi.org/10.31083/AP43531.
