# Supplementary material for: Altered Expression of GABA-Related Genes in Schizophrenia: Insights from Meta-Analyses of Brain and Blood Samples and iPSC-Derived Organoids
Source: Alpha Psychiatry. 2026 Feb 25;27(1):43531. doi: 10.31083/AP43531 (PMC12957970; doi:10.31083/AP43531)
Supplement: Supplementary file 1 [file 2757-8038-27-1-43531-s1.zip › PRISMA_checklist.docx]

| **Section and Topic** | **Item #** | **Checklist item** | **Location where item is reported** |
| --- | --- | --- | --- |
| **TITLE** | | |  |
| Title | 1 | Identify the report as a systematic review. | Page 1 – Title page  As described in the title, we performed a systematic meta-analysis  (and not a systematic review) |
| **ABSTRACT** | | |  |
| Abstract | 2 | See the PRISMA 2020 for Abstracts checklist. | Page 1 – Abstract section |
| **INTRODUCTION** | | |  |
| Rationale | 3 | Describe the rationale for the review in the context of existing knowledge. | As described in the introduction (page 1): while previous findings regarding GAD1 expression were consistent, there are inconsistencies regarding the GAD2 gene expression in tissue samples of subjects with schizophrenia. Moreover, the interplay between these genes remains unclear |
| Objectives | 4 | Provide an explicit statement of the objective(s) or question(s) the review addresses. | In the last paragraph of the introduction (page 2):   1. We systematically calculate the differential expression of GAD1, GAD2, as well as SST and PVALB, and their correlation patterns in different brain regions of patients with schizophrenia. 2. We examined their expression in organoids derived from patients, offering insights into earlier neurodevelopmental stages, and in blood samples from patients to assess their potential as biomarkers. |
| **METHODS** | | |  |
| Eligibility criteria | 5 | Specify the inclusion and exclusion criteria for the review and how studies were grouped for the syntheses. | From the first section in Methods (page 3): Inclusion criteria were set and strictly followed for dataset selection: human schizophrenia versus control study of post-mortem Brodmann area 10, Brodmann area 22/superior temporal gyrus, cerebellum samples, parietal cortex, anterior cingulate cortex, nucleus accumbens, striatum or hippocampus samples, comparable conditions and availability of gene expression preprocessed data. Exclusion criteria: datasets that were duplicates, datasets that are not comparable with most of the rest of the datasets in terms of global differential expression (methodology explained below), and datasets with less than seven samples of patients with schizophrenia, were excluded. |
| Information sources | 6 | Specify all databases, registers, websites, organisations, reference lists and other sources searched or consulted to identify studies. Specify the date when each source was last searched or consulted. | From the first section in Methods (page 3): Publicly available gene expression datasets were searched in three public repositories: the National Center for Biotechnology Information (NCBI) Gene Expression Omnibus (GEO) (<http://www.ncbi.nlm.nih.gov/geo/>) and the Stanley Medical Research Institute (SMRI) Array Collection (<http://www.stanleyresearch.org/brain-research/array-collection/>). |
| Search strategy | 7 | Present the full search strategies for all databases, registers and websites, including any filters and limits used. | From the first section in Methods (page 3): The following keywords were used: Schizophrenia, gene expression, human, and brain samples. Figure 1 presents the complete  workflow of eligible dataset selection |
| Selection process | 8 | Specify the methods used to decide whether a study met the inclusion criteria of the review, including how many reviewers screened each record and each report retrieved, whether they worked independently, and if applicable, details of automation tools used in the process. | Page 3 – Method section  Assif Yitzhaky performed the database search described above, and following the inclusion criteria decided whether a study met the criteria. |
| Data collection process | 9 | Specify the methods used to collect data from reports, including how many reviewers collected data from each report, whether they worked independently, any processes for obtaining or confirming data from study investigators, and if applicable, details of automation tools used in the process. | Page 3 – Method section  Gene expression datasets and sample characteristics were downloaded by Assif Yitzhaky. Preprocessing methods for each of  the datasets are described in the supplementary information file. |
| Data items | 10a | List and define all outcomes for which data were sought. Specify whether all results that were compatible with each outcome domain in each study were sought (e.g. for all measures, time points, analyses), and if not, the methods used to decide which results to collect. | Outcomes for which data were sought:  GAD1, GAD2, SST, and PVALB expression levels in each of  the patients of each of the datasets included in the meta-analysis, as described in the Results section (page 5). |
|  | 10b | List and define all other variables for which data were sought (e.g. participant and intervention characteristics, funding sources). Describe any assumptions made about any missing or unclear information. | Additional variables: sample type, platform, number of cases and controls, gender, age, post-mortem interval (PMI), pH and gene expression data for the relevant genes ([Table 3](https://www.ncbi.nlm.nih.gov/pmc/articles/PMC5125005/table/t1/))  as described in the Methods section (page 4).  Estimation of the effect of potential confounding factors  Datasets for which this information was not available were not  included in the relevant analysis. |
| Study risk of bias assessment | 11 | Specify the methods used to assess risk of bias in the included studies, including details of the tool(s) used, how many reviewers assessed each study and whether they worked independently, and if applicable, details of automation tools used in the process. | Page 3 – Method section  Assif Yitzhaky and Libi Hertzberg accessed each study. However, it  should be noted that they did not work independently. The decision  on which study gene expression data is included in the meta-  analysis has been made without any knowledge regarding the actual gene expression results for the genes we study in this meta-analysis (GAD1, GAD2, SST and PVALB).  This fact minimizes the risk for biases, such as selection bias,  publication bias (we included studies that did not publish the results  for these genes) |
| Effect measures | 12 | Specify for each outcome the effect measure(s) (e.g. risk ratio, mean difference) used in the synthesis or presentation of results. | As described in Methods (page 4), we used Effect size (Hedges’ g), which is the standardized difference between the expression in the disease vs. control samples. |
| Synthesis methods | 13a | Describe the processes used to decide which studies were eligible for each synthesis (e.g. tabulating the study intervention characteristics and comparing against the planned groups for each synthesis (item #5)). | For a given gene, all studies which measured this gene’s expression were included in the synthesis. Quality control and pre-processing steps are described in the Supplementary information file. |
|  | 13b | Describe any methods required to prepare the data for presentation or synthesis, such as handling of missing summary statistics, or data conversions. | Quality control and pre-processing steps that were applied for each  of the datasets included in the meta-analysis are described in the  Supplementary information file. Regarding missing values: All four genes were measured in all the datasets included in the meta-analysis (see Figures 2, 4) and there were no missing values |
|  | 13c | Describe any methods used to tabulate or visually display results of individual studies and syntheses. | As described in the results section (page 5), Forest plots were generated using the function “forest” from the “meta” package in R, version 4.9- 2 (General Package for Meta-Analysis). |
|  | 13d | Describe any methods used to synthesize results and provide a rationale for the choice(s). If meta-analysis was performed, describe the model(s), method(s) to identify the presence and extent of statistical heterogeneity, and software package(s) used. | Meta-analysis: As described in the results section (page 5), Effect size (Hedges’ g), which is the standardized difference between the  expression in the disease vs. control samples, was calculated  separately for each of the datasets. Hedges’g and confidence  interval values were calculated for each of the datasets using the  function “metacont” from the “meta” package in R, a general package for meta-analysis, version 4.9-2. To address the differences in study design, platform usage and the heterogeneity existing among microarray datasets, we applied the Effect size (ES) combination with Random Effect Modeling, which takes both the direction and magnitude of gene expression changes into consideration to generate more biologically consistent results (Fleiss, 1993) |
|  | 13e | Describe any methods used to explore possible causes of heterogeneity among study results (e.g. subgroup analysis, meta-regression). | As described in the supplementary information file, we filtered out outlier samples. |
|  | 13f | Describe any sensitivity analyses conducted to assess robustness of the synthesized results. | As described in the results section (page 5), we performed correlation analysis between the expression of GAD1, GAD2, PVALB and SST genes, to give an additional measure for the validity and the robustness of the meta-analysis results |
| Reporting bias assessment | 14 | Describe any methods used to assess risk of bias due to missing results in a synthesis (arising from reporting biases). | As seen in Figures 2 and 4 in the results section, the four genes were measured in all the datasets included in the meta-analysis and there were no missing results. |
| Certainty assessment | 15 | Describe any methods used to assess certainty (or confidence) in the body of evidence for an outcome. | As described in the results section (page 5), we performed correlation analysis between the expression of GAD1 and GAD2, to give an additional measure for the validity and the robustness of the meta- analysis results.  In addition, as described in the discussion section, we performed a  literature survey and estimated the consistency between our results  and previous relevant studies. |
| **RESULTS** | | |  |
| Study selection | 16a | Describe the results of the search and selection process, from the number of records identified in the search to the number of studies included in the review, ideally using a flow diagram. | A flow diagram of the search and selection process of the studies  included in the meta-analysis is given in Figure 1. |
|  | 16b | Cite studies that might appear to meet the inclusion criteria, but which were excluded, and explain why they were excluded. | Studies that met the inclusion criteria but were excluded due to  duplications are listed and cited in the supplementary information file. |
| Study characteristics | 17 | Cite each included study and present its characteristics. | Each of the study’s characteristics are described in the  supplementary information file |
| Risk of bias in studies | 18 | Present assessments of risk of bias for each included study. | The decision on which study gene expression data is included in the meta-analysis has been made without any knowledge regarding the actual gene expression results for the genes we study in this meta- analysis (GAD1, GAD2, SST and PVALB). This fact minimizes the risk for biases, such as selection bias, publication bias (we included studies that did not publish the  results for these genes). We did not apply further assessments of the risk of bias for each of the included studies. |
| Results of individual studies | 19 | For all outcomes, present, for each study: (a) summary statistics for each group (where appropriate) and (b) an effect estimate and its precision (e.g. confidence/credible interval), ideally using structured tables or plots. | (a) Summary statistics are given in Table 3  (b) Effect estimate and confidence interval are given in Figure  2, Figure 4 |
| Results of syntheses | 20a | For each synthesis, briefly summarise the characteristics and risk of bias among contributing studies. | The characteristics of the contributing studies are summarised in  Table 3. |
|  | 20b | Present results of all statistical syntheses conducted. If meta-analysis was done, present for each the summary estimate and its precision (e.g. confidence/credible interval) and measures of statistical heterogeneity. If comparing groups, describe the direction of the effect. | The results are presented in Figure 2 and Figure 4 |
|  | 20c | Present results of all investigations of possible causes of heterogeneity among study results. | Page 4 – Method section  To address the differences in study design, platform usage and the heterogeneity existing among microarray datasets, we applied the Effect size (ES) combination with Random Effect Modeling, which takes both the direction and magnitude of gene expression changes into consideration to generate more biologically consistent results (Fleiss, 1993) |
|  | 20d | Present results of all sensitivity analyses conducted to assess the robustness of the synthesized results. | We did not perform sensitivity analyses. |
| Reporting biases | 21 | Present assessments of risk of bias due to missing results (arising from reporting biases) for each synthesis assessed. | As seen in Figures 2 and 4 in the results section, the four genes were measured in all the datasets included in the meta-analysis and there were no missing results. |
| Certainty of evidence | 22 | Present assessments of certainty (or confidence) in the body of evidence for each outcome assessed. | As described in the results section (page 5), we performed a correlation analysis between the expression of GAD1, GAD2, SST and PVALB, to give an additional measure for the validity and the robustness of the meta-analysis results. |
| **DISCUSSION** | | |  |
| Discussion | 23a | Provide a general interpretation of the results in the context of other evidence. | As described in the discussion section (page 5), we performed a literature survey and estimated the consistency between our results and previous relevant studies. We discussed any inconsistency and gave relevant interpretation. |
|  | 23b | Discuss any limitations of the evidence included in the review. | We listed the limitations of the studies included in the meta-analysis, in the discussion section (page 8). |
|  | 23c | Discuss any limitations of the review processes used. | We listed the limitations of the meta-analysis we performed, in the  discussion section (page 8). |
|  | 23d | Discuss implications of the results for practice, policy, and future research. | We discussed implications of the results for practice and future  research, in the discussion section (page 10). |
| **OTHER INFORMATION** | | |  |
| Registration and protocol | 24a | Provide registration information for the review, including register name and registration number, or state that the review was not registered. | None. |
|  | 24b | Indicate where the review protocol can be accessed, or state that a protocol was not prepared. | None. |
|  | 24c | Describe and explain any amendments to information provided at registration or in the protocol. | None. |
| Support | 25 | Describe sources of financial or non-financial support for the review, and the role of the funders or sponsors in the review. | None. |
| Competing interests | 26 | Declare any competing interests of review authors. | None. |
| Availability of data, code and other materials | 27 | Report which of the following are publicly available and where they can be found: template data collection forms; data extracted from included studies; data used for all analyses; analytic code; any other materials used in the review. | All the data used for all the analysis are publicly available, as  described in the Methods section and in the supplementary  information file. |

*From:*  Page MJ, McKenzie JE, Bossuyt PM, Boutron I, Hoffmann TC, Mulrow CD, et al. The PRISMA 2020 statement: an updated guideline for reporting systematic reviews. BMJ 2021;372:n71. doi: 10.1136/bmj.n71. This work is licensed under CC BY 4.0. To view a copy of this license, visit <https://creativecommons.org/licenses/by/4.0/>
