# Supplementary material for: Altered Expression of GABA-Related Genes in Schizophrenia: Insights from Meta-Analyses of Brain and Blood Samples and iPSC-Derived Organoids
Source: Alpha Psychiatry. 2026 Feb 25;27(1):43531. doi: 10.31083/AP43531 (PMC12957970; doi:10.31083/AP43531)
Supplement: Supplementary file 1 [file 2757-8038-27-1-43531-s1.zip › Supplementary Material.docx]

Altered Expression of GABA-Related Genes in Schizophrenia: Insights from Meta-Analyses of Brain and Blood Samples and iPSC-Derived Organoids

GABA-Genes: 3-Layer Analysis in Schizophrenia

SUPPLEMENTARY INFORMATION

Yuval Singer^1^, Assif Yitzhaky^2^, Libi Hertzberg^1,2,3,^*

^1^School of Medicine, Gray Faculty of Medical & Health Sciences, Tel Aviv University, 69978, Tel Aviv, Israel

^2^Department of Physics of Complex Systems, Weizmann Institute of Science, 7610001 Rehovot, Israel

^3^Shalvata Mental Health Center, 45100 Hod Hasharon, Israel

*Correspondence: libi.hertzberg@gmail.com (Libi Hertzberg)

CONTENTS

[LIST OF FIGURES 3](#_Toc208309593)

[LIST OF TABLES 3](#_Toc208309594)

[BRAIN SAMPLES DATASETS CHARACTERISTICS 4](#_Toc208309595)

[**Maycox 2009 BA10 dataset** 4](#_Toc208309596)

[**Pietersen 2014 Pyramidal STG dataset** 4](#_Toc208309597)

[**Paz 2006 cerebellum dataset** 5](#_Toc208309598)

[**Chen 2013 parietal cortex dataset** 5](#_Toc208309599)

[**Ramaker 2017 anterior cingulate gyrus (AnCg) dataset** 6](#_Toc208309600)

[**Lanz 2019 hippocampus dataset** 6](#_Toc208309601)

[**Perez 2021 hippocampal subfield CA3 dataset** 7](#_Toc208309602)

[INDUCED PLURIPOTENT CELLS (iPSCs)-DERIVED DATASETS CHARACTERISTICS 7](#_Toc208309603)

[**Kathuria 2020 iPSC-derived cerebral organoid dataset** 7](#_Toc208309604)

[BLOOD SAMPLES DATASETS CHARACTERISTICS 8](#_Toc208309605)

[**Bousman et al. 2010, GEO accession: GSE18312** 8](#_Toc208309606)

[**van Beveren et al. 2012 dataset** 8](#_Toc208309607)

[**De Jong et al. 2012 dataset** 9](#_Toc208309608)

[SELECTION OF ELIGIBLE GENE EXPRESSION DATASETS FOR PARTICIPANT-DATA META-ANALYSIS 10](#_Toc208309609)

[SUPPLEMENTARY FIGURES AND TABLES 11](#_Toc208309610)

[BIBLIOGRAPHY 13](#_Toc208309611)

# LIST OF FIGURES

[Supplementary Fig. 1. -Log2 of p-values of Pairwise Pearson correlation of the four genes along schizophrenia samples in the datasets included in the meta-analysis. The color in each entry (x,y) represents the –Log2 of the p-value associated with the Pearson correlation coefficient between the expression of the gene in row x and the gene in column y, measured along the schizophrenia samples. Gene symbols are ordered in the same order in both the x- and the y-axes. 11](#_Toc208309612)

# LIST OF TABLES

[Supplementary Table 1. Linear model t-statistic values and associated p-values for each of the seven datasets and each of the four genes. T-statistic values higher than zero are marked red and t-statistic values lower than zero are marked blue. The color intensity is proportional to the t-statistic value. The p-value associated with each t-statistic value appears in the row below the t-statistic value. 12](#_Toc220335564)

[Supplementary Table 2. Blood samples expression meta-analysis results. The standardized difference (Random effects (44) is negative (blue) if the expression is lower in schizophrenia vs. the control group and positive (red) if the expression is higher in the control group vs. schizophrenia. The color intensity is proportional to the Random effects Hedges value. The lower and upper limits of the 95% confidence interval are given in the 4th and 5th columns. The associated p-values are given in 6th column. I², τ², and Cochran’s Q test and its associated p-value are given in columns 7th-10th. 12](#_Toc220335565)

# BRAIN SAMPLES DATASETS CHARACTERISTICS

## **Maycox 2009 BA10 dataset**

The dataset GDS4523 (Maycox et al., 2009) was downloaded from the GEO database (<https://www.ncbi.nlm.nih.gov/sites/GDSbrowser?acc=GDS4523>). The dataset consists of 51 BA10 brain samples from subjects with schizophrenia (n=28) and healthy controls (n=23). See Fig. 2 for samples’ characteristics. Samples were run on Affymetrix Human Genome U133 Plus 2.0 Arrays. **Normalization method:** Arrays were scanned on a GeneChip Scanner 3000 and fluorescence intensity for each feature of the array was obtained by using GeneChip Operating Software (Affymetrix) (Maycox et al., 2009). As described in GSE17612_series_matrix.txt (available at <https://www.ncbi.nlm.nih.gov/geo/query/acc.cgi?acc=GSE17612>), the data were analyzed with Microarray Suite version 5.0 (MAS 5.0) using Affymetrix default analysis settings and global scaling as normalization method. The trimmed mean target intensity of each array was set to 150. We then applied threshold and log2. The threshold value was determined using scatter plots of healthy control samples in order to estimate the noise level (the threshold after log2 that was used is 4). **Outlier removal:** Outlier samples removal was applied similarly to (Oldham et al., 2008). Shortly, the following steps were applied: 1) Pearson correlation coefficient values between each pair of samples were calculated. 2) For each sample, the mean correlation with all other sample was calculated. 3) The mean, M, and standard deviation, S, of all samples’ mean values (calculated in 2.) were calculated. 4) Samples whose mean correlation (found in 2.) were smaller than M-3*S were considered as outliers and were removed from the rest of the analysis. One schizophrenia sample was removed. After outlier removal, 50 samples were left (27 schizophrenia and 23 controls). **Filtering**: The initial number of probe-sets was 54,613. 1) In case of a gene symbol with multiple probe-sets, the probe-set with the highest mean expression over the samples was taken into account and the other probe-sets were discarded. Number of genes after this step: 30,805. 2) Genes that were absent (values equal or lower than the threshold) in more than 70% of both the schizophrenia and the control samples, were filtered out. Number of genes after filtering: 27,261.

##

## **Pietersen 2014 Pyramidal STG dataset**

The dataset GSE37981 (Pietersen et al., 2014) was downloaded from the GEO database (<https://www.ncbi.nlm.nih.gov/geo/query/acc.cgi?acc=GSE37981>). The initial dataset consists of 18 brain samples isolated from pyramidal cells in layer III of the superior temporal gyrus (STG) with schizophrenia (n=9) and healthy controls (n=9). The samples were run on Affymetrix Human X3P Array. **Normalization method:** “Each array was scanned twice and the Affymetrix Microarray Suite 5.1 software averaged the two images to compute an intensity value for each probe cell within each probe set” (Pietersen et al., 2014).

We then applied threshold and log2. The threshold value was determined using scatter plots of healthy control samples in order to estimate the noise level (the threshold after log2 that was used is 3).  **Outlier removal:** Outlier samples removal was applied similarly to (Oldham et al., 2008) and as described above. After outlier removal, 1 normal sample was removed. After the removal, the dataset consists of 17 samples (9 schizophrenia and 8 controls). **Filtering**: Initial number of probe-sets: 61,359 (48,860 with gene symbols). 1) In case of a gene symbol with multiple probe-sets, the probe-set with the highest mean expression over the samples was taken into account and the other probe-sets were discarded. Number of genes after this step: 21,606. 2) Genes that were absent (values equal or lower than the threshold) in more than 70% of both the schizophrenia and the control samples, were filtered out. Number of genes after filtering: 17,886.

## **Paz 2006 cerebellum dataset**

The dataset GDS1917 (Paz et al., 2006) was downloaded from the GEO database (<https://www.ncbi.nlm.nih.gov/sites/GDSbrowser?acc=GDS1917>). The initial dataset consists of 28 brain samples corresponding to the crus I/VIIa area of the cerebellum from subjects with schizophrenia (n=14) and healthy controls (n=14) (The samples were obtained from the Maryland Brain Collection). The samples were run on Affymetrix Human Genome U133 Plus 2.0 Array. **Normalization method:** We applied threshold and Log2 transformation. The threshold value was determined using scatter plots of healthy control samples in order to estimate the noise level (the threshold after Log2 that was used is 4). **Outlier removal:** Outlier samples removal was applied similarly to (Oldham et al., 2008). Shortly, the following steps were applied: 1) Pearson correlation coefficient values between each pair of samples were calculated. 2) For each sample, the mean correlation with all other sample was calculated. 3) The mean, M, and standard deviation, S, of all samples’ mean values (calculated in 2.) were calculated. 4) Samples whose mean correlation (found in 2.) were smaller than M-3*S were considered as outliers and were removed from the rest of the analysis. One schizophrenia sample was removed. After the removal, 27 samples were left (13 schizophrenia and 14 controls). **Filtering**: The initial number of probe-sets was 54,613. 1) In case of a gene symbol with multiple probe-sets, the probe-set with the highest mean expression over the samples was taken into account and the other probe-sets were discarded. Number of genes after this step: 30,803. 2) Genes that were absent (values equal or lower than the threshold) in more than 70% of both the schizophrenia and the control samples, were filtered out. Number of genes after filtering: 27,329.

##

## **Chen 2013 parietal cortex dataset**

The dataset GSE35978 (Chen et al., 2013) was downloaded from the GEO database (<https://www.ncbi.nlm.nih.gov/geo/query/acc.cgi?acc=GSE35978>). The initial dataset consisted of 312 brain samples, including subjects with schizophrenia (n=51) and healthy controls (n=50) from the parietal cortex. Samples were run on Affymetrix Human Gene 1.0 ST Array [transcript (gene) version]. **Normalization method:** “The data were analyzed by RMA using Affymetrix Expression Console with default analysis settings” (described in GSE35978_series_matrix.txt, available at the aforementioned link). After applying RMA, no threshold or log2 are needed. That is because the RMA normalization method includes the following steps: 1. Background correction 2. Quantile normalization 3. Log2 transformation.

**Outlier removal:** Outlier samples removal was applied similarly to (Oldham et al., 2008) and as described above. (**Note**: this procedure was performed on the initial dataset of 312 samples). 5 control samples were removed. After the removal, the dataset consists of 51 schizophrenia samples and 45 healthy control samples. **Filtering**: The initial number of probe-sets was 33,297 (25,293 with gene symbols). In case of a gene symbol with multiple probe-sets, the probe-set with the highest mean expression over the samples (of both brain regions) was taken into account and the other probe-sets were discarded. Number of genes after this step: 23,307.

##

## **Ramaker 2017 anterior cingulate gyrus (AnCg) dataset**

The dataset GSE80655 (Ramaker et al., 2017) was downloaded from the GEO database (<https://www.ncbi.nlm.nih.gov/geo/query/acc.cgi?acc=GSE80655>). The initial dataset consisted of 281 brain samples, including subjects with schizophrenia (n=24) and healthy controls (n=24) from the anterior cingulate gyrus (AnCg). Samples were run on Illumina HiSeq 2000 (Homo sapiens). **Normalization method:** “RNA-seq paired-end FASTQ files were aligned to hg19 using Tophat and Cufflinks. Read counts for each transcript were compiled across all post-mortem tissues samples” (described in GSE80655_series_matrix.txt, available at the aforementioned link). We then applied threshold and log2. The threshold value was determined using scatter plots of healthy control samples in order to estimate the noise level (the threshold after log2 that was used is 3). **Outlier removal:** Outlier samples removal was applied similarly to (Oldham et al. 2008) and as described above. (**Note:** this procedure was performed on the initial dataset of 281 samples). 1 schizophrenia sample was removed. After the removal, the dataset consists of 23 schizophrenia samples and 24 healthy control samples. **Filtering**: Initial number of probe-sets: 57,905 (52,442 with gene symbols). 1) In case of a gene symbol with multiple probe-sets, the probe-set with the highest mean expression over the samples (of both brain regions) was taken into account and the other probe-sets were discarded. Number of genes after this step: 51,310. 2) Genes that were absent (values equal or lower than the threshold) in more than 70% of both the schizophrenia and the control samples, were filtered out. Number of genes after filtering: 23,175.

##

## **Lanz 2019 hippocampus dataset**

The dataset GSE53987 (Lanz et al. 2019) was downloaded from the GEO database (<https://www.ncbi.nlm.nih.gov/geo/query/acc.cgi?acc=GSE53987>). The initial dataset consisted of 205 brain samples, including subjects with schizophrenia (n=15) and healthy controls (n=18) from the hippocampus. Samples were run on Affymetrix Human Genome U133 Plus 2.0 Arrays. **Normalization method:** “R/Bioconductor was used to apply the Robust Multi-Array Average (RMA) methodology to generate expression values. Brain regions were normalized separately” (described in GSE53987_series_matrix.txt, available at the aforementioned link). We then applied log2. **Outlier removal:** Outlier samples removal was applied similarly to (Oldham et al., 2008) and as described above. (**Note**: this procedure was performed on the initial dataset of 205 samples). No sample was removed. **Filtering**: The initial number of probe-sets was 54,613 (45,772 with gene symbols). In case of a gene symbol with multiple probe-sets, the probe-set with the highest mean expression over the samples (of both brain regions) was taken into account and the other probe-sets were discarded. Number of genes after this step: 22,880.

## **Perez 2021 hippocampal subfield CA3 dataset**

The dataset GSE138082 (Perez et al. 2021) was downloaded from the GEO database (<https://www.ncbi.nlm.nih.gov/geo/query/acc.cgi?acc=GSE138082>). The initial dataset consisted of 78 brain samples, including subjects with schizophrenia (n=39 (13 samples from CA3)) and healthy controls (n=39 (13 from CA3)). Samples were run on Illumina HiSeq 2500 Arrays. **Normalization method:** Single-end/Paired-end reads were aligned with STAR 2.5.2b to the reference sequence (hg19 from UCSC). Human reference was used for gene annotation and counting (GRCh37.87). Uniquely mapped reads were quantified using HTSeq python suite using protein coding genes by gene (described in GSE138082_series_matrix.txt, available at the aforementioned link). We then applied threshold and log2. The threshold value was determined using scatter plots of healthy control samples in order to estimate the noise level (the threshold after log2 that was used is 5). **Outlier removal:** Outlier samples removal was applied similarly to (Oldham et al. 2008) and as described above. (**Note**: this procedure was performed on the initial dataset of 78 samples). One control sample (CA3) was removed. After the removal, the dataset consists of 13 schizophrenia samples and 12 healthy control samples from CA3. **Filtering:** The initial number of gene symbols was 20,268. Genes that were absent (values equal or lower than the threshold) in more than 70% of both the schizophrenia and the control samples in CA3, were filtered out. Number of genes after filtering: 14,571.

##

# INDUCED PLURIPOTENT CELLS (iPSCs)-DERIVED DATASETS CHARACTERISTICS

## **Kathuria 2020 iPSC-derived cerebral organoid dataset**

The dataset GSE133534 (van Beveren et al., 2012) was downloaded from the GEO database (<https://www.ncbi.nlm.nih.gov/geo/query/acc.cgi?acc=GSE133534>). The initial dataset consists of 21 induced Pluripotent Stem Cell (iPSC) derived cerebral organoids samples with schizophrenia (n=10) and healthy controls (n=11). Samples were run on Illumina NovaSeq 6000 (Homo sapiens). **Normalization method:** Sequence quality was assessed using FASTQC. Sequences were trimmed using cutadapt v 1.18. Trimmed reads were aligned to the reference genome using TopHat v2.1.1. Bam files were merged using samptools v 1.9 for samples with techincal replicates. FPKM calculation done using cufflinks v2.2.1. (described in GSE133534_series_matrix.txt, available at the aforementioned link). After merging the replicates, the dataset consists of 16 samples (8 schizophrenia and 8 controls). We then applied threshold and log2. The threshold value was determined using scatter plots of healthy control samples in order to estimate the noise level (the threshold after log2 that was used is log2(0.008)). **Outlier removal:** Outlier samples removal was applied similarly to (Oldham et al., 2008) and as described above. No sample was removed. **Filtering**: The initial number of gene symbols was 49,554. 1) In case of identical symbols, the symbol with the highest mean expression over the samples was taken into account and the other identical symbols were discarded. Number of gene symbols after this step: 45,331. 2) Genes that were absent (values equal or lower than the threshold) in more than 70% of both the schizophrenia and the control samples, were filtered out. Number of gene symbols after filtering: 34,302.

# BLOOD SAMPLES DATASETS CHARACTERISTICS

## **Bousman et al. 2010, GEO accession: GSE18312**

The dataset GSE18312 (Bousman et al., 2010) was downloaded from the GEO database (<https://www.ncbi.nlm.nih.gov/geo/query/acc.cgi?acc=GSE18312>). The original dataset consisted of 30 whole blood samples, including subjects with schizophrenia (n=13) and healthy controls (n=8). **Platform:** Affymetrix Human Exon 1.0 ST Array [transcript (gene) version]. **Normalization method:**  “The data were analyzed with Partek Genomics Suite v6.4 using RMA to analyze probe intensities and to determine differential gene expression between diagnostic and control groups” (described in GSE18312_series_matrix.txt, available at the aforementioned link). **Outlier removal:** Outlier samples removal was applied similarly to (Oldham et al., 2008) and as described above. (**Note**: this procedure was performed on the initial dataset of 30 samples). 1 schizophrenia sample was removed. After the removal, the dataset consists of 12 schizophrenia samples and 8 healthy control samples. **Filtering**: The initial number of gene symbols was 17,634. In case of a gene symbol with multiple probe-sets, the probe-set with the highest mean expression over the samples was taken into account and the other probe-sets were discarded. Number of genes after this step: 17,324.

## **van Beveren et al. 2012 dataset**

The dataset GSE27383 (van Beveren et al., 2012) was downloaded from the GEO database (<https://www.ncbi.nlm.nih.gov/geo/query/acc.cgi?acc=GSE27383>). The dataset consists of 72 Peripheral Blood Mononuclear Cells (PBMCs) samples from subjects with schizophrenia (n=43) and healthy controls (n=29). Samples were run on Affymetrix Human Genome U133 Plus 2.0 Arrays. **Normalization method:** GeneChips were scanned using the Hewlett-Packard GeneArray Scanner G2500A. Raw intensities values of all samples were normalized by RMA normalization (Robust Multichip Analysis) (background correction and quantile normalization) using Partek version 6.4 (described in GSE27383_series_matrix.txt, available at the aforementioned link). **Outlier removal:** Outlier samples removal was applied similarly to (Oldham et al., 2008) and as described above. 2 schizophrenia samples were removed. After the removal, the dataset consists of 41 schizophrenia samples and 29 healthy control samples. **Filtering**: The initial number of probe-sets was 54,613 (45,772 with gene symbols). In case of a gene symbol with multiple probe-sets, the probe-set with the highest mean expression over the samples was taken into account and the other probe-sets were discarded. Number of genes after this step: 22,880.

## **De Jong et al. 2012 dataset**

The dataset GSE38484 (de Jong et al., 2012) was downloaded from the GEO database (<https://www.ncbi.nlm.nih.gov/geo/query/acc.cgi?acc=GSE38484>). The dataset consists of 202 whole blood samples from subjects with schizophrenia (n=106) and healthy controls (n=96). Samples were run on Illumina HumanHT-12 V3.0 expression beadchip. **Normalization method:** Data were background corrected in Beadstudio (v. 3.2.3) and transformed (vst) and normalized (rsn) in Lumi (R package) (described in GSE38484_series_matrix.txt, available at the aforementioned link). **Outlier removal:** Outlier samples removal was applied similarly to (Oldham et al., 2008) and as described above. Two schizophrenia samples were removed. After the removal, the dataset consists of 104 schizophrenia samples and 96 healthy control samples. **Filtering**: The initial number of probe-sets was 48,743 (36,108 with gene symbols). In case of a gene symbol with multiple probe-sets, the probe-set with the highest mean expression over the samples was taken into account and the other probe-sets were discarded. Number of genes after this step: 25,142.

# SELECTION OF ELIGIBLE GENE EXPRESSION DATASETS FOR PARTICIPANT-DATA META-ANALYSIS

Publicly available gene expression datasets were searched in two public repositories: NCBI Gene Expression Omnibus (GEO) (http://www.ncbi.nlm.nih.gov/geo/) and the Stanley Medical Research Institute (SMRI) Array Collection (http://www.stanleyresearch.org/brain-research/array-collection/). The following keywords and their combinations were used: “schizophrenia” AND (”human ”OR “homo sapiens”) (”gene expression” OR “mRNA” OR messenger RNA”) AND (”cerebellum” OR ”Brodmann Area 10” OR  “BA10” OR ”Brodmann Area 22” OR “BA22” OR ”superior temporal gyrus”  OR ”cyngulate cortex” OR ”parietal cortex” OR “nucleus accumbens” OR “striatum” OR “hippocampus”). We note that we did not include datasets from the dorsolateral prefrontal cortex. Given the large number of available datasets from this region, including them in accordance with PRISMA guidelines would have required incorporating all of them, which was beyond the scope of the present work.

The following information was extracted from each of the datasets included in our participant-data meta-analysis: sample type, platform, number of cases and controls, PubMed ID and the preprocessed gene expression data. The inclusion criteria were: 1) Schizophrenia versus control study of post-mortem brain samples, 2) Brain samples from one of the brain regions listed above. 3) Availability of whole-genome gene expression preprocessed data. Exclusion criteria: 1) Duplicates of included datasets. The full datasets' characteristics are described in the Supplementary Information.

# SUPPLEMENTARY FIGURES AND TABLES


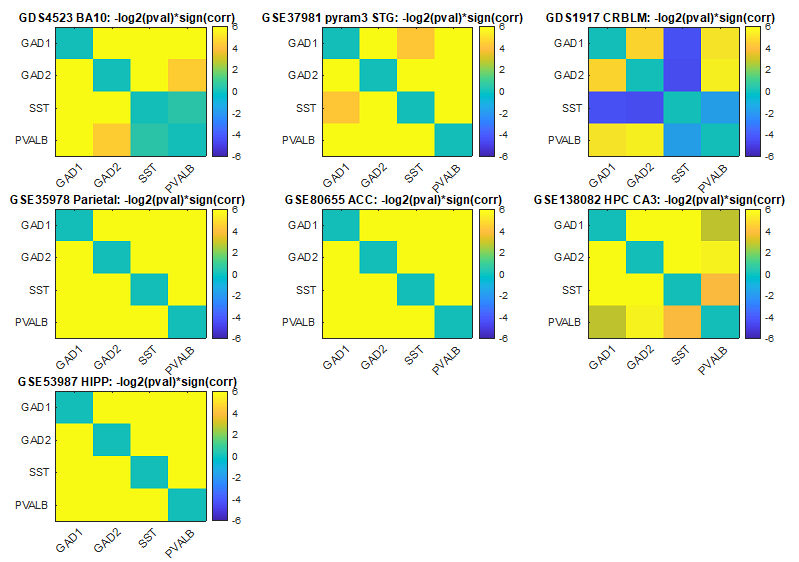
Supplementary Fig. 1. -Log2 of p-values of Pairwise Pearson correlation of the four genes along schizophrenia samples in the datasets included in the meta-analysis. The color in each entry (x,y) represents the –Log2 of the p-value associated with the Pearson correlation coefficient between the expression of the gene in row x and the gene in column y, measured along the schizophrenia samples. Gene symbols are ordered in the same order in both the x- and the y-axes.

Supplementary Table 1. Linear model t-statistic values and associated p-values for each of the seven datasets and each of the four genes. T-statistic values higher than zero are marked red and t-statistic values lower than zero are marked blue. The color intensity is proportional to the t-statistic value. The p-value associated with each t-statistic value appears in the row below the t-statistic value.

| # | **Dataset** | **GAD1** | **GAD2** | **SST** | **PVALB** | **Covariates included** |
| --- | --- | --- | --- | --- | --- | --- |
| 1 | GDS4523 BA10 t-stat | 1.83 | 1.74 | 0.39 | 0.35 | Age, Gender, PMI, pH |
|  | p-value | 0.07 | 0.09 | 0.69 | 0.73 |  |
| 2 | GSE37981 pyram3 STG t-stat | -0.12 | -0.84 | -0.49 | -0.99 | Age, Gender, PMI, RIN, pH |
|  | p-value | 0.91 | 0.42 | 0.63 | 0.35 |  |
| 3 | GDS1917 CRBLM t-stat | -1.72 | -0.51 | -1.42 | 0.05 | Age, Gender, PMI |
|  | p-value | 0.10 | 0.62 | 0.17 | 0.96 |  |
| 4 | GSE35978 parietal t-stat | -1.63 | -1.12 | -1.59 | -1.50 | Age, Gender, Lifetime Anti-psychotics, PMI, pH |
|  | p-value | 0.11 | 0.27 | 0.12 | 0.14 |  |
| 5 | GSE80655 ACC t-stat | -2.85 | -3.02 | -3.59 | 0.24 | Age, Gender, PMI, pH |
|  | p-value | 0.01 | 0.00 | 0.00 | 0.81 |  |
| 6 | GSE53987 HPC t-stat | -1.36 | -2.36 | -2.77 | -2.32 | Age, Gender, PMI, RIN, pH |
|  | p-value | 0.19 | 0.03 | 0.01 | 0.03 |  |
| 7 | GSE138082 HPC CA3 t-stat | -0.27 | -0.82 | -0.63 | -0.44 | Age, Gender, PMI, RIN |
|  | p-value | 0.79 | 0.42 | 0.54 | 0.67 |  |
|  | **Weighted mean t-statistic** | -1.01 | -0.98 | -1.54 | -0.74 |  |

**Supplementary Table 2. Blood samples expression meta-analysis results.** The standardized difference (Random effects (44) is negative (blue) if the expression is lower in schizophrenia vs. the control group and positive (red) if the expression is higher in the control group vs. schizophrenia. The color intensity is proportional to the Random effects Hedges value. The lower and upper limits of the 95% confidence interval are given in the 4th and 5th columns. The associated p-values are given in 6th column. I², τ², and Cochran’s Q test and its associated p-value are given in columns 7th-10th.

| **#** | **Gene symbol** | **Random effects Hedges** | **Lower** | **Upper** | **p-value** | **tau** | **I2** | **Q** | **Q p-value** |
| --- | --- | --- | --- | --- | --- | --- | --- | --- | --- |
| 1 | GAD1 | -0.04 | -0.27 | 0.19 | 0.74 | 0 | 0 | 1.7 | 0.44 |
| 2 | GAD2 | -0.05 | -0.44 | 0.35 | 0.82 | 0.24 | 0.48 | 3.9 | 0.14 |
| 3 | PVALB | -0.26 | -0.5 | -0.03 | 0.027 | 0 | 0 | 0.61 | 0.74 |
| 4 | SST | -0.05 | -0.28 | 0.18 | 0.68 | 0 | 0 | 1.5 | 0.47 |

# BIBLIOGRAPHY

Bousman, C.A., Chana, G., Glatt, S.J., Chandler, S.D., Lucero, G.R., Tatro, E., May, T., Lohr, J.B., Kremen, W.S., Tsuang, M.T., Everall, I.P., 2010. Preliminary evidence of ubiquitin proteasome system dysregulation in schizophrenia and bipolar disorder: convergent pathway analysis findings from two independent samples. Am. J. Med. Genet. B. Neuropsychiatr. Genet. 153B, 494–502. https://doi.org/10.1002/ajmg.b.31006

Chen, C., Cheng, L., Grennan, K., Pibiri, F., Zhang, C., Badner, J.A., Kelsoe, J.R., Greenwood, T.A., Nievergelt, C.M., Barrett, T.B., McKinney, R., Shilling, P.D., Schork, N.J., Smith, E.N., Bloss, C.S., Nurnberger, J., Edenberg, H.J., Foroud, T., Koller, D.L., Scheftner, W., Lawson, W.B., Nwulia, E.A., Hipolito, M., Coryell, W., Rice, J., Byerley, W., McMahon, F., Chen, D.T.W., Schulze, T.G., Berrettini, W., Potash, J.B., Zandi, P.P., Mahon, P.B., McInnis, M., Craig, D., Szelinger, S., Gershon, E.S., Liu, C., 2013. Two gene co-expression modules differentiate psychotics and controls. Mol. Psychiatry 18, 1308–1314. https://doi.org/10.1038/mp.2012.146

de Jong, S., Boks, M.P.M., Fuller, T.F., Strengman, E., Janson, E., de Kovel, C.G.F., Ori, A.P.S., Vi, N., Mulder, F., Blom, J.D., Glenthøj, B., Schubart, C.D., Cahn, W., Kahn, R.S., Horvath, S., Ophoff, R.A., 2012. A gene co-expression network in whole blood of schizophrenia patients is independent of antipsychotic-use and enriched for brain-expressed genes. PLoS One 7. https://doi.org/10.1371/JOURNAL.PONE.0039498

Maycox, P.R., Kelly, F., Taylor, A., Bates, S., Reid, J., Logendra, R., Barnes, M.R., Larminie, C., Jones, N., Lennon, M., Davies, C., Hagan, J.J., Scorer, C.A., Angelinetta, C., Akbar, T., Hirsch, S., Mortimer, A.M., Barnes, T.R.E., de Belleroche, J., de Belleroche, J., 2009. Analysis of gene expression in two large schizophrenia cohorts identifies multiple changes associated with nerve terminal function. Mol. Psychiatry 14, 1083–1094. https://doi.org/10.1038/mp.2009.18

Oldham, M.C., Konopka, G., Iwamoto, K., Langfelder, P., Kato, T., Horvath, S., Geschwind, D.H., 2008. Functional organization of the transcriptome in human brain. Nat. Neurosci. 11, 1271–1282. https://doi.org/10.1038/nn.2207

Paz, R.D., Andreasen, N.C., Daoud, S.Z., Conley, R., Roberts, R., Bustillo, J., Perrone-Bizzozero, N.I., 2006. Increased Expression of Activity-Dependent Genes in Cerebellar Glutamatergic Neurons of Patients With Schizophrenia. Am. J. Psychiatry 163, 1829–1831. https://doi.org/10.1176/ajp.2006.163.10.1829

Pietersen, C.Y., Mauney, S.A., Kim, S.S., Lim, M.P., Rooney, R.J., Goldstein, J.M., Petryshen, T.L., Seidman, L.J., Shenton, M.E., McCarley, R.W., Sonntag, K.C., Woo, T.U.W., 2014. Molecular profiles of pyramidal neurons in the superior temporal cortex in schizophrenia. J. Neurogenet. 28, 53–69. https://doi.org/10.3109/01677063.2014.882918

Ramaker, R.C., Bowling, K.M., Lasseigne, B.N., Hagenauer, M.H., Hardigan, A.A., Davis, N.S., Gertz, J., Cartagena, P.M., Walsh, D.M., Vawter, M.P., Jones, E.G., Schatzberg, A.F., Barchas, J.D., Watson, S.J., Bunney, B.G., Akil, H., Bunney, W.E., Li, J.Z., Cooper, S.J., Myers, R.M., 2017. Post-mortem molecular profiling of three psychiatric disorders. Genome Med. 9, 72. https://doi.org/10.1186/s13073-017-0458-5

van Beveren, N.J.M., Buitendijk, G.H.S., Swagemakers, S., Krab, L.C., Röder, C., de Haan, L., van der Spek, P., Elgersma, Y., 2012. Marked reduction of AKT1 expression and deregulation of AKT1-associated pathways in peripheral blood mononuclear cells of schizophrenia patients. PLoS One 7. https://doi.org/10.1371/JOURNAL.PONE.0032618
